# Reaction Model and Mechanism of Preparing (Al_2_O_3_ + C) Precursor for Carbothermal Synthesis of AlN by a Modified Low Temperature Combustion Synthesis Method

**DOI:** 10.3390/ma15186216

**Published:** 2022-09-07

**Authors:** Aimin Chu, Longqing Zhang, Rafi Ud-din, Yuping Zhao

**Affiliations:** 1 School of Materials Science and Engineering, Hunan University of Science and Technology, Xiangtan 411201, China; 2 Materials Division, PINSTECH, Post Office Nilore, Islamabad 44000, Pakistan; 3 School of Civil and Engineering, Hunan University of Science and Technology, Xiangtan 411201, China

**Keywords:** (Al_2_O_3_ + C) precursor, reaction model and mechanism, modified low temperature combustion synthesis, nitrogen-containing gases, nanomaterials

## Abstract

The preparation of a homogeneous mixture of (Al_2_O_3_ + C) precursor is the key step for the successful synthesis of AlN powders by the carbothermal reduction and nitridation method. In the present work, the homogeneous (Al_2_O_3_ + C) precursor prepared by a modified low temperature combustion synthesis (MLCS) method by using aluminum nitrate, glucose, and urea as materials exhibited high reaction activity. Furthermore, in order to absolutely control the MLCS process and continuously improve the properties of (Al_2_O_3_ + C) precursor, the reaction model of preparing precursors from various molar ratios of urea to aluminum nitrate (U/Al) was investigated by carrying out thermodynamic calculation and by performing experiments in the present work. The whole process was found to involve various phenomena. First, the type and amount of various generated nitrogen-containing gases (N_2_, NO, N_2_O, N_2_O_3_, N_2_O_4_, and NO_2_) vary with the change of U/Al during combustion process. Second, under the present experimental condition of ignition temperature, the decomposition reaction of aluminum nitrate is more prone to occur than the combustion reaction of urea. Third, the real reaction system with U/Al = 2.5 reaches the highest combustion temperature which is well consistent with the propellant chemical theory. The occurrence of above phenomena was discussed in detail. Moreover, the reaction mechanism of synthesizing precursor from U/Al = 1 with high reaction activity was investigated by using various techniques such as FTIR, XRD, and DTA.

## 1. Introduction

Low temperature combustion synthesis (LCS), invented in the mid-1980s, is an outstanding approach for the preparation of nanoscale materials [1]. The LCS method is rapidly emerging as one of the most convenient methods for the preparation of oxide materials [2,3]. During recent years, LCS has gained wide acceptance due to its excellent characteristics. Firstly, a large amount of gas is released in the reaction process to produce a strong dispersion effect preventing powder agglomeration. Secondly, combustion heat completes the reaction instantly to avoid component segregation as well as to yield the uniform combustion powder product with high specific surface area and high activity. Thirdly, it is a simple and low-cost process which is conducive to industrial production.

Due to above-mentioned advantages, LCS as a new preparation method for nano-powder material has been widely employed and studied since its discovery [4,5,6]. However, most researchers studied the LCS as a preparation method for nano oxide ceramic powders [7]. During recent years, research reports about preparation of metal and alloy powders using LCS method have also been documented [8,9,10]. For the first time, our research team have proposed the research idea of preparing non-oxide ceramic powder by combining a modified LCS (MLCS) and carbothermal reduction and nitridation (CRN) methods. MLCS has the advantages of wet chemistry, which ensures the precise control of the uniform mixing and proportion of components in the liquid phase. Moreover, compared with the traditional wet chemistry (such as LCS method, sol-gel method, precipitation method, etc.), MLCS also exhibits unique advantages as follows [11,12,13,14,15]. Firstly, the combustion releases a lot of heat and gas, transforming the metal salt into fine metal oxide particles, and evenly dispersed in the fine carbon particles formed by the decompose of carbon source to prevent component segregation. Secondly, the absorption of heat arising from the decomposing of the carbon source can decrease the combustion temperature, and the released gas arising from the decomposed carbon source can enhance the dispersion effect of product particles. These facilitate the preparation of amorphous combustion powder with smaller particle size, more uniform dispersion, and higher reactivity.

This MLCS method has been employed to prepare various nanometer non-oxide ceramic powders such as CrN, WC, AlN, etc. [11,12,13]. This MLCS-CRN method is generally comprised of three steps (such as for preparing AlN nano-powder) as follows [14,15]. First, soluble organic carbon source (glucose, sucrose, citric acid, etc.) is added to the mixed solution of oxidant (such as aluminum nitrate) and fuel (such as urea), which changes the combustion mechanism and phenomenon resulting in uniform and fine Al_2_O_3_ + C precursor. Then, this precursor has been used to synthesize AlN + C product by CRN method. Finally, the AlN + C product is calcined at 700 °C in air for 1 h to move the residual carbon and to obtain pure AlN nano-powder. Especially for the preparation of AlN nano-powder, various research works have been carried out and many meaningful results have also been reported by our research team recently [14,15]. Moreover, it is well known that the proper selection of raw materials and the preparation of a homogeneous mixture of (Al_2_O_3_ + C) precursors are the two essential tasks needed to accomplish the successful synthesis of AlN powders by the CRN method [13,16].

However, during our research regarding the preparation of AlN powders by MLCS-CRN method, a regular variation in the particle size and morphology of precursors and CRN products has been observed with increasing (U/Al). Moreover, the solution containing glucose with U/Al = 1 can prepare the (Al_2_O_3_ + C) precursor with high reaction activity [14,15]. Furthermore, no significant systematic studies have been conducted to investigate the reaction model and mechanism of preparing (Al_2_O_3_ + C) precursor. Therefore, in the present work, in order to control absolutely the MLCS process and continuously improve the properties of (Al_2_O_3_ + C) precursor, firstly, the various U/Al contents were employed for the synthesis of (Al_2_O_3_ + C) precursors via this MLCS route; secondly, the reaction model of preparing precursors from various molar ratios of U/Al was investigated by carrying out thermodynamic calculation and by performing experiments. Furthermore, the effect of U/Al on the type and amount of generating nitrogen-containing gases is discussed in detail. Moreover, the reaction mechanism of preparing (Al_2_O_3_ + C) precursor with high reaction activity was also investigated systematically.

## 2. Materials and Methods

Three analytical reagents of glucose (C_6_H_12_O_6_·H_2_O), urea (CO(NH_2_)_2_), and aluminum nitrate (Al(NO_3_)_3_·9H_2_O) were used as raw materials to prepare (Al_2_O_3_ + C) precursor powders by MLCS method. The molar ratio of urea to aluminum nitrate (U/Al) in solution was in the range of 0–4. The molar ratio of glucose to aluminum nitrate (C/Al) in solution was fixed at 8. The concentration of aluminum nitrate was 0.1 mol in solution.

The procedure of preparing (Al_2_O_3_ + C) precursor was carried out according to our previous documents [14,15]. The combustion temperature of the solution system was measured by a nickel–chromium (K type) thermocouple. The phase studies of samples were performed by using X-ray diffraction analysis (XRD, Rigaku (Tokyo, Japan), D/max-RB12). Differential thermal analysis of samples was performed in air up to 300 °C at a heating rate of 5 °C min^−1^ by using a thermal analyzer (DTA, Rigaku, DT-40). The Fourier transform infrared spectra (FT-IR) were recorded using a Nicolet 740 FT-IR spectrometer.

## 3. Results

### 3.1. Thermodynamic Analysis of Generating Various Nitrogen-Containing Gases

Generally, according to the propellant chemical theory, the reaction of the conventional LCS method between urea and aluminum nitrate is shown in Reaction (1) [14]. When the U/Al is 2.5, the maximum energy can be released in the reaction system. At this stage, the combustion temperature is the highest and the nitrogen-containing gas generated is only considered to be N_2_.


(1) $$2\mathrm{Al}{\left({\mathrm{NO}}_{3}\right)}_{3}+5\mathrm{CO}{\left({\mathrm{NH}}_{2}\right)}_{2}\to {\mathrm{Al}}_{2}O_{3}+5{\mathrm{CO}}_{2}↑+10H_{2}O+8N_{2}↑$$

In fact, the combustion system containing glucose with U/Al = 1 synthesized the (Al_2_O_3_ + C) precursor with best characteristics in our previous research results [14,15]. Therefore, in the combustion system added with glucose with U/Al = 1, in addition to N_2_, the other nitrogen-containing gases including NO, N_2_O, N_2_O_3_, N_2_O_4_, and NO_2_ may also be produced. The exothermic reaction between urea and aluminum nitrate or the decomposition reaction of glucose will occur simultaneously during heating. Therefore, in this experiment, the above two type reactions generating various nitrogen-containing gases will be merged. These reactions generating various nitrogen-containing gases can be expressed by Reactions (2)–(7) as follows:

(2) $$\mathrm{Al}{\left({\mathrm{NO}}_{3}\right)}_{3}\cdot 9H_{2}O+\frac{5}{2}\mathrm{CO}{\left({\mathrm{NH}}_{2}\right)}_{2}+\frac{8}{6}C_{6}H_{12}O_{6}\to \frac{1}{2}{\mathrm{Al}}_{2}O_{3}+\frac{5}{2}{\mathrm{CO}}_{2}+22H_{2}O+4N_{2}+8C$$

(3) $$\mathrm{Al}{\left({\mathrm{NO}}_{3}\right)}_{3}\cdot 9H_{2}O+\frac{9}{10}\mathrm{CO}{\left({\mathrm{NH}}_{2}\right)}_{2}+\frac{8}{6}C_{6}H_{12}O_{6}\to \frac{1}{2}{\mathrm{Al}}_{2}O_{3}+\frac{9}{10}{\mathrm{CO}}_{2}+\frac{104}{5}H_{2}O+\frac{24}{5}\mathrm{NO}+8C$$

(4) $$\mathrm{Al}{\left({\mathrm{NO}}_{3}\right)}_{3}\cdot 9H_{2}O+\frac{3}{2}\mathrm{CO}{\left({\mathrm{NH}}_{2}\right)}_{2}+\frac{8}{6}C_{6}H_{12}O_{6}\to \frac{1}{2}{\mathrm{Al}}_{2}O_{3}+\frac{3}{2}{\mathrm{CO}}_{2}+20H_{2}O+3N_{2}O+8C$$

(5) $$\mathrm{Al}{\left({\mathrm{NO}}_{3}\right)}_{3}\cdot 9H_{2}O+\frac{1}{2}\mathrm{CO}{\left({\mathrm{NH}}_{2}\right)}_{2}+\frac{8}{6}C_{6}H_{12}O_{6}\to \frac{1}{2}{\mathrm{Al}}_{2}O_{3}+\frac{1}{2}{\mathrm{CO}}_{2}+18H_{2}O+2N_{2}O_{3}+8C$$

(6) $$\mathrm{Al}{\left({\mathrm{NO}}_{3}\right)}_{3}\cdot 9H_{2}O+\frac{3}{14}\mathrm{CO}{\left({\mathrm{NH}}_{2}\right)}_{2}+\frac{8}{6}C_{6}H_{12}O_{6}\to 7{\mathrm{Al}}_{2}O_{3}+\frac{3}{14}{\mathrm{CO}}_{2}+\frac{122}{7}H_{2}O+\frac{12}{7}N_{2}O_{4}+8C$$

(7) $$\mathrm{Al}{\left({\mathrm{NO}}_{3}\right)}_{3}\cdot 9H_{2}O+\frac{1}{2}\mathrm{CO}{\left({\mathrm{NH}}_{2}\right)}_{2}+\frac{8}{6}C_{6}H_{12}O_{6}\to \frac{1}{2}{\mathrm{Al}}_{2}O_{3}+\frac{1}{2}{\mathrm{CO}}_{2}+18H_{2}O+4{\mathrm{NO}}_{2}+8C$$

In order to check the feasibility of Reactions (2)–(7) to occur under certain conditions, the Gibbs free energy change (ΔG) of these reactions was calculated, and then the occurring tendency of these reactions was analyzed according to the result. The enthalpy of material is calculated by using Equations (8) and (9) as follows:

(8) $$\Delta H_{298K}^{\theta }=\sum^{\text{​}}\vartheta_{i}\Delta_{f}H^{\theta }\left(i,\;298K\right)\;$$

(9) $$\Delta H_{T}^{\theta }=\Delta H_{298K}^{\theta }+\int^{\text{​}}\Delta C_{P}dT\;$$

The entropy of materials is calculated by using Equations (10) and (11) as follows:

(10) $$\Delta S_{298K}^{\theta }=\sum^{\text{​}}\vartheta_{i}S^{\theta }\left(i,\;298K\right)\;$$

(11) $$\Delta S_{T}^{\theta }=\Delta S_{298K}^{\theta }+\int^{\text{​}}\frac{\Delta C_{P}}{T}dT\;$$

The ΔG of Reactions (2)–(7) is calculated by using Equation (12) as follows:

(12) $$\Delta G_{T}=\Delta H_{T}-T\Delta S_{T}\;$$

*Cp* in Equation (11) refers to the thermal capacity of reactants and resultants in constant pressure, T0 is 298 K, and reaction temperature (T) is 300–1200 K according to actual condition. All thermodynamic data of reactants and resultants were obtained from the JANAF tables [17].

**Figure 1:**
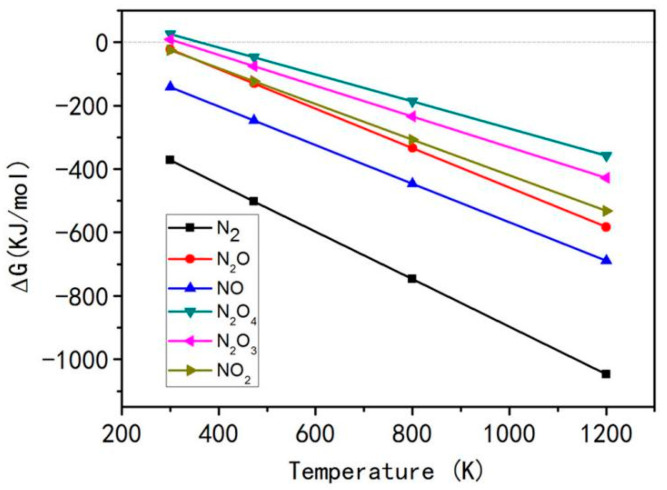
ΔG of Equations (2)–(7) for formation of various nitrogen-containing gases.

The Gibbs free energy change (ΔG) of Reactions (2)–(7) is calculated by using Equations (8)–(12). The ΔG results of Reactions (2)–(7) are shown in Figure 1. The igniting temperature of combustion system is 350 °C (623 K). As shown in Figure 1, the ΔG values of Reactions (2)–(7) of generating various nitrogen-containing gases in the range of 473–1200 K, were less than zero, indicating that the solution system consisting of urea, aluminum nitrate, and glucose can generate various nitrogen-containing gases (i.e., Reactions (2)–(7)) during the combustion reaction. Next, the type and amount of nitrogen-containing gases generated in the combustion systems with various U/Al contents will be analyzed in detail.

### 3.2. Analysis of Generating Nitrogen-Containing Gases under Various U/Al

It is obvious from Reactions (3)–(7) that the amount of urea needed in these reactions is less than that required in Reaction (2). Moreover, of all these reactions, the Reaction (6) requires the minimum amount of urea needed for generating N_2_O_4_ (U/Al = 3/14). Moreover, the Reactions (2)–(7) can occur simultaneously in the combustion system with U/Al = 1. If only the Reaction (2) predominates in the combustion system with U/Al = 1, the amount of urea will be insufficient resulting in the decomposition of the excessive aluminum nitrate to produce red brown NO_2_ gas directly during combustion reaction. However, in the present experiment, no red brown gas is found, suggesting that in the combustion system, not only the reaction of generating N_2_ (Reaction (2)) occurred, but also other reactions (Reactions (3)–(7)) that need lesser amount of urea took place.

Moreover, no red brown NO_2_ gas was found in the combustion systems with U/Al > 1 (such as 1.5), indicating non-occurrence of Reaction (7). Nevertheless, Figure 1 shows that the reaction tendency of generating NO_2_ gas (Reaction (7)) is stronger than those of N_2_O_3_ (Reaction (5)) and N_2_O_4_ (Reaction (6)). This result suggests that the Reactions (5) and (6) cannot occur in the combustion reaction systems with U/Al > 1. Therefore, aside from N_2_, other two nitrogen-containing gases i.e., NO (Reaction (3)) and N_2_O (Reaction (4)) can produce during combustion reaction. When the igniting temperature is 350 °C, the ΔG for generation of NO and N_2_O gases is less than zero (Figure 1). Moreover, the U/Al of these two reactions is close to 1, suggesting that the reactions of generating NO and N_2_O gases cannot be ignorant in the combustion system.

When the U/Al ˂ 1 (such as 0.5), the evolution of red brown NO_2_ gas is observed to be taken place during the process of combustion reaction. There are three ways of generating NO_2_ gas. First is the decomposition of excessive aluminum nitrate under igniting temperature, as shown in Reaction (13) [18]. The second one is by Reaction (7). The third is obtained from other nitrogen-containing gases (NO, N_2_O, N_2_O_3_, and N_2_O_4_) which are transformed into NO_2_ gas, since these unstable nitrogen-containing gases are very prone to transform into NO_2_ gas in air.


(13) $$4\mathrm{Al}{\left({\mathrm{NO}}_{3}\right)}_{3}\cdot 9H_{2}O\to 2{\mathrm{Al}}_{2}O_{3}+12{\mathrm{NO}}_{2}+3O_{2}+36H_{2}O$$

Only heating of the urea will decompose urea to generate HCNO and NH_3_ (Reaction (14)). Nevertheless, if heating with aluminum nitrate is conducted, then the generated gases of combustion system will be the mixture comprising of H_2_O, CO_2_, N_2_, HCNO, and NH_3_ according to Reactions (14)–(16) [19]. In fact, the type and amount of releasing final gases are mainly dependent upon the available amount of internal oxygen from Reaction (13) and the U/Al (as shown in Reactions (2)–(7)).

(14) $$\mathrm{CO}{\left({\mathrm{NH}}_{2}\right)}_{2}\to {\mathrm{NH}}_{3}+\mathrm{HCNO}$$

(15) $$\mathrm{HCNO}+1/2O_{2}\to 1/3N_{2}+1/3{\mathrm{NH}}_{3}+CO_{2}$$

(16) $${\mathrm{NH}}_{3}+3/4O_{2}\to 1/2N_{2}+3/2H_{2}O$$

It is reported that in the presence of urea, the decomposition and combustion reactions of aluminum nitrate are synchronous [18]. This implies that by only heating the aluminum nitrate, the red brown NO_2_ gas will generate. However, if aluminum nitrate reacts with a moderate amount of urea, the NO_2_ gas will not be generated in combustion system, as shown in Reaction (2). Therefore, in the presence of a moderate amount of urea, the decomposition reaction of aluminum nitrate and the combustion reaction of urea can be expressed by Reactions (13) and (17), respectively [20].

(17) $${\mathrm{CO}({\mathrm{NH}}_{2})}_{2}+1.5O_{2}={\mathrm{CO}}_{2}+2H_{2}O+N_{2}$$

**Figure 2:**
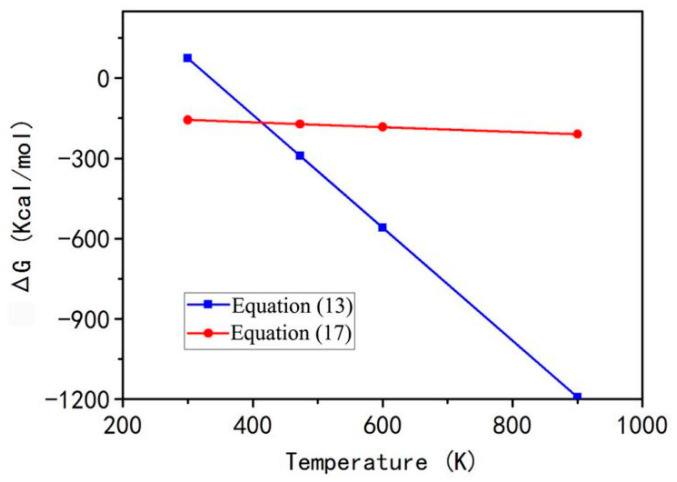
ΔG for various reaction temperatures for Equations (13) and (17).

Based on the thermodynamic data of JANAF tables [17], the ΔG of Reactions (13) and (17) is calculated by using Equations (8)–(12), and the corresponding results are shown in Figure 2. It is obvious from Figure 2 that the ΔG of two reactions is less than zero under the ignition temperature of 350 °C. Moreover, the decomposition reaction of aluminum nitrate can more easily take place than the combustion reaction of urea above 425 K. At about 152 °C (425 K), the curves of two reactions intersect representing two reactions to occur simultaneously. In fact, the ignition temperature of the combustion system is 350 °C (623 K), which facilitates the reaction between aluminum nitrate and urea.

**Figure 3:**
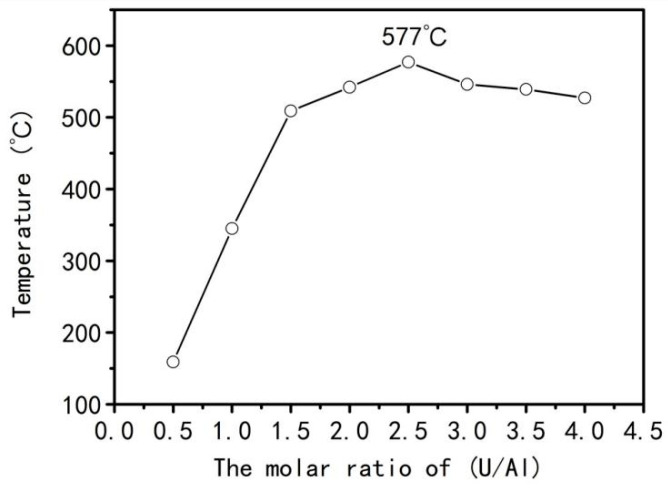
Combustion temperature of the solutions with various (U/Al).

Therefore, the above results have indicated that the red brown NO_2_ gas can be generated by employing Reactions (3)–(7) with the U/Al contents less than 1. In addition, the real combustion temperatures in combustion systems with various (U/Al) contents are measured by experiment (Figure 3). It is obvious from Figure 3 that when the value of U/Al reaches 2.5, the combustion temperature is the highest (about 577 °C), which is in accordance with the propellant chemical theory [14]. However, when the value of U/Al is greater than 2.5, the combustion temperature decreases slightly due to the generated gas from the decomposition of excessive urea utilizing a part of heat. However, the combustion temperature still holds above 500 °C, indicating the influence of decomposition of excessive urea on the combustion temperature is finite.

The reaction between urea and aluminum nitrate is an exothermic reaction according to Reaction (17) [20]. Under the condition of sufficient oxygen, the greater amount of urea will result in greater heat release, and consequently the combustion temperature of system will be higher. However, in the present experiment, the actual combustion temperature of system reached the maximum when U/Al was 2.5. With an increase of urea amount, the combustion temperature of the system decreased slightly, as shown in Figure 3. It is reported that urea can only react with oxygen (namely internal oxygen) arising from the decomposition of aluminum nitrate. The oxygen in air (namely external oxygen) is not involved in the reaction with urea, which only reacts with carbon rooting in the decomposition of glucose [19,21]. The results of Figure 2 indicate that the decomposition of urea and aluminum nitrate takes place and the redox reactions between urea and aluminum nitrate are synchronous. However, the above results have shown that 1 mol aluminum nitrate needs 2.5 mol urea (U/Al = 2.5) to react completely. If the U/Al is more than 2.5, the excessive amount of urea cannot obtain enough oxygen from aluminum nitrate to react with them and release heat. Instead, excessive urea needs to absorb heat to decompose and release lot of gases and hence utilizing a part of heat from the combustion system resulting in a decrease in the combustion temperature. This analysis results are consistent with the combustion temperatures shown in Figure 3.

When the amount of urea is excessive (i.e., U/Al > 2.5), the amount of generating oxygen from the corresponding Reaction (13) is not sufficient; therefore, Reaction (17) is complete to some extent and results in release of a lot of NH_3_ gas out of air. In fact, in this study, we also verified that the released gas is NH_3_ by testing the PH of product gas and smelling its odor, indicating the generated NH_3_ is arising from the decomposition of an excessive amount of urea. Thus, when the amount of urea is excessive (i.e., U/Al > 2.5), the reactions among aluminum nitrate, glucose, and urea correspond to Reactions (2) and (14). Therefore, it is clear from above discussion that when the U/Al is more than 2.5, the Reaction (2) that generates N_2_ is dominant. Moreover, the decomposition reaction of excessive amount of urea (Reaction (14)) also arises synchronously.

### 3.3. Reaction Mechanism of Preparing (Al_2_O_3_ + C) Precursor with High Reactivity

It is documented in our previous reports that (Al_2_O_3_ + C) precursor with good reactivity can be prepared by the MLCS method using U/Al = 1 [14,15]. Compared with the precursors prepared from other U/Al contents, the (Al_2_O_3_ + C) precursor from U/Al = 1 can significantly improve the characteristics of the CRN resulting in AlN nanopowder with fine particle size and uniform distribution. In the following section, the MLCS process of solution will be further studied in order to reveal the reaction mechanism for the preparation of the (Al_2_O_3_ + C) precursor with high reactivity.

Chandramouli et al. [22] have found that the solution of thorium nitrate and urea will form a transition complex between two reactants during heating by LCS method. The molecular formula is Th(NO_3_)_4_·xCO(NH_2_)_2_·yH_2_O, where x is usually 4 or 6, and y is 4 or 2. The specific value depends on the preparation method of the complex. It is clear from this literature that the amount of urea required for 1 mol of nitrate is usually greater than that required for 4 moles in the complex formed by nitrate and urea alone. Kim et al. [23] synthesized an intact sugar metal complex (C_9_H_18_FeNO_8_) with D-glucose (C_6_H_12_O_6_), chloride (FeCl_2_), and alanine (CH_3_CH(NH_2_)CO_2_H). Further, it was also verified that another complex (namely Ala/Glu), formed between alanine and glucose, was also detected. Moreover, it is also reported in our previous work that the precursor with high activity can be prepared by appropriate urea addition (such as U/Al = 1) by MLCS method, while the precursor prepared without or with more urea has exhibited poor activity [14].

From above-mentioned results, it can be concluded that the addition of glucose affects the molecular structure and type of the complex formed in the solution. Moreover, following the addition of glucose, various complexes may be formed among glucose, aluminum nitrate, and urea in our work, and these complexes will change the combustion process as well as the characteristics of the combustion products. The reaction mechanism of the mixed solution with glucose may be quite different from that of the solution without organic carbon source (such as glucose). Therefore, it is necessary to further study the combustion process and reaction mechanism of the solution with organic carbon source.

**Figure 4:**
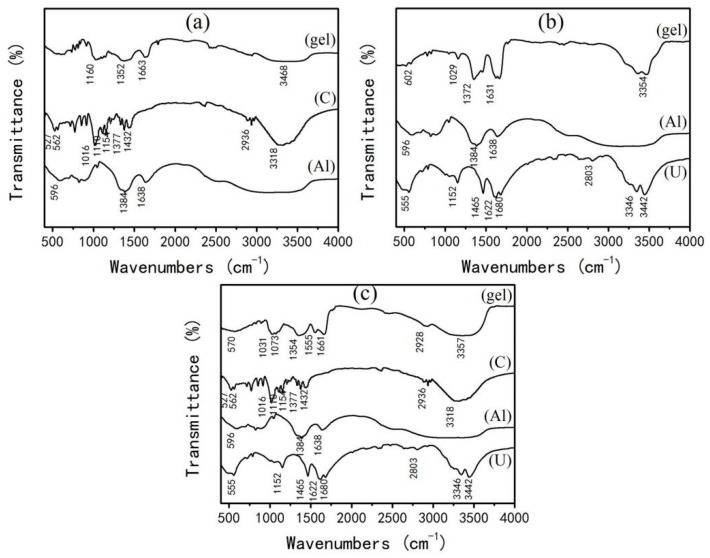
IR spectra of the gels and related materials: (**a**) gel of glucose and aluminum nitrate (C/Al = 8); (**b**) gel of urea and aluminum nitrate (U/Al = 1); (**c**) gel of urea, glucose, and aluminum nitrate (U/Al = 1, C/Al = 8).

From the above analysis results, it can be concluded that complex may be formed between urea and aluminum nitrate, and among urea and aluminum nitrate and glucose. Therefore, in this study, three possible complexes formed in the solution with various materials are characterized so as to disclose the reaction mechanism of the solution. Figure 4a show the IR spectra of the gel formed by glucose (C) and aluminum nitrate (Al) (C/Al = 8). It can be seen from Figure 4a that compared with raw materials (glucose and aluminum nitrate), several main peaks (such as 1663, 1352, and 1160 cm^−1^) of the gel causing certain deviations, and two other peaks 1432 and 1154 cm^−1^ of glucose disappeared, indicating that a new complex formed in the gel [24]. Figure 4b shows the IR spectra of the gel formed between urea and aluminum nitrate (U/Al = 1). It can be seen that the gel exhibits certain peaks which are not present in spectra of raw materials. For example, peaks of 1631, 1372, 1029, and 602 cm^−1^ are not found in spectra of the raw materials, and the characteristic peak of urea at 1680 cm^−1^ disappears in the formed gel. All these phenomena indicate that a new complex is formed in the gel. Figure 4c shows the IR spectra of the gel formed among urea, aluminum nitrate, and glucose (U/Al = 1, C/Al = 8). It can be seen that the gel has a certain deviation from multiple peaks of raw materials, indicating that a new complex is formed in the gel.

From above mentioned results it can be concluded that in the solution, complexes with various decomposition temperatures are formed among urea, aluminum nitrate, and glucose, namely, the complex formed among urea, aluminum nitrate, and glucose, the complex formed between urea and aluminum nitrate, and the complex formed between aluminum nitrate and glucose.

**Figure 5:**
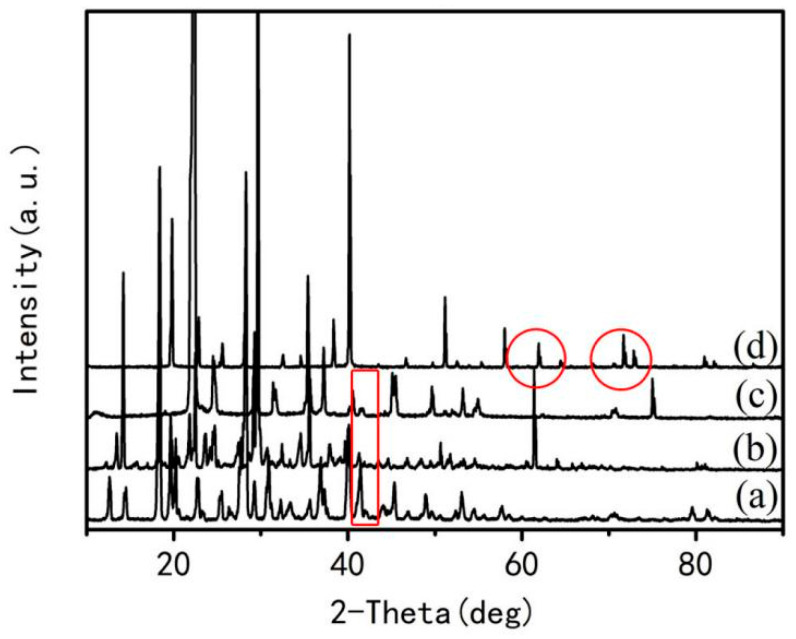
XRD spectra of the materials and gel: (**a**) glucose; (**b**) aluminum nitrate; (**c**) urea; (**d**) gel.

**Figure 6:**
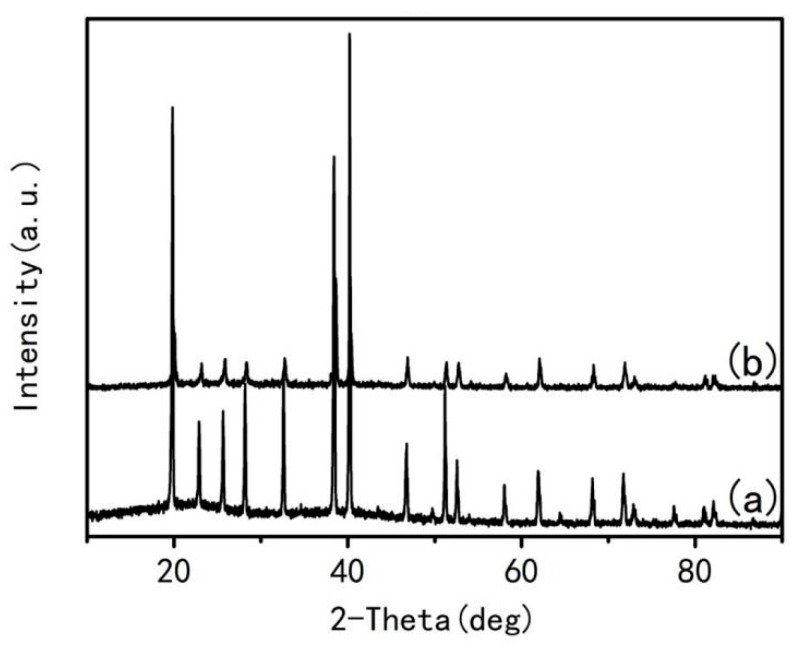
XRD spectra of gels under different conditions: (**a**) before foam; (**b**) before combustion reaction.

In order to further confirm these complexes formed in these gels, all raw materials and the dried gels were analyzed by XRD and the results are shown in Figure 5. It can be seen from Figure 5 that diffraction peaks arising from different raw materials appearing in the gel are different, some new diffraction peaks appear in the gel (indicated by ring in Figure 5). Moreover, there is no simple overlap spectra between three raw materials and the gel (indicated by square in Figure 5), indicating that the aqueous solution consisting of glucose, aluminum nitrate, and urea forms a new complex during heating. The diffraction intensities for the complex before combustion reaction are much lower than that of the expansion (before foaming) in the previous period, as shown in Figure 6, indicating that the complex has been partially decomposed. However, the crystalline phases of XRD peaks in Figure 6 have not been discriminated by Jade software, indicating that these transition phases generated under special conditions have not been employed by the Jade database.

**Figure 7:**
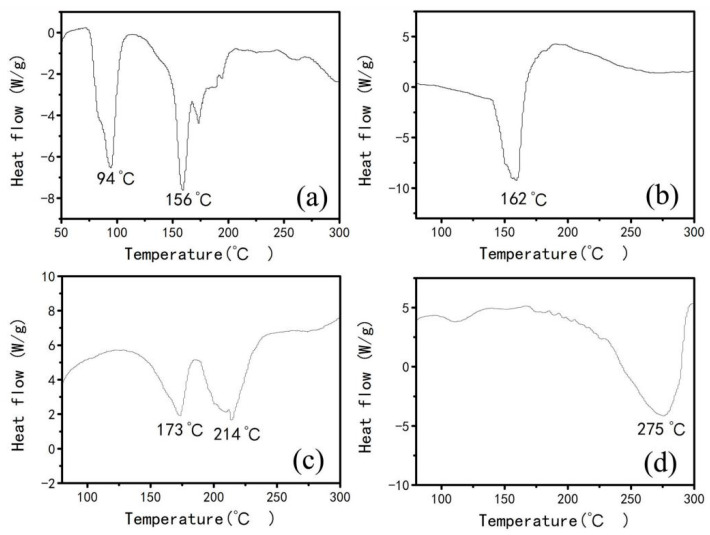
DTA curves of various substances: (**a**) glucose; (**b**) complex formed between glucose and aluminum nitrate (C/Al = 8); (**c**) complex formed among glucose, urea, and aluminum nitrate (C/Al = 8, U/Al = 1); (**d**) complex formed between urea and aluminum nitrate (U/Al = 1).

From the results of Figure 4, Figure 5 and Figure 6, it is clear that reaction among glucose, urea, and aluminum nitrate can form various complexes. Subsequently, differential thermal analysis (DTA) of these complexes is carried out to understand their thermal stability. Figure 7 shows the DTA spectra of glucose and various complexes. It can be seen from Figure 7 that the thermal decomposition temperature of the complex, formed between urea and aluminum nitrate (Figure 7d), was higher than that of the complex formed between glucose and aluminum nitrate (Figure 7b), and the complex formed among glucose, urea, and aluminum nitrate (Figure 7c). Moreover, the thermal decomposition temperatures of three complexes are higher than that of the glucose alone (Figure 7a). It is also obvious that the thermal decomposition temperature of the complex, formed between urea and aluminum nitrate, is the highest (275 °C) among four samples and indicates its relatively high stability. Therefore, in our experiment, four substances with different decomposition temperatures may exist in the gel formed by glucose, urea, and aluminum nitrate (U/Al = 1). As shown in Figure 7, the decomposition temperatures of four substances gradually increase in a gradient state, indicating their decomposition behavior one by one.

During the heating process, multiple competitive reactions occur in the mixed solution of glucose, urea and aluminum nitrate. The target reaction is the complex reaction between metal ions and complexing agent. At the same time, the crystallization reactions of metal ions and other anions such as nitrate ions take place during the rapid evaporation and reduction of solvent at higher temperature. If the strong complexing agent (urea) in the solution is too small, it cannot effectively prevent the crystallization and precipitation of aluminum nitrate, resulting in compositional inhomogeneity distribution in the prepared precursor. However, excess urea may form a complex with high thermal decomposition temperature, so that the gel does not decompose during the previous process, and thus releasing gas and expand during heating, resulting in the prepared precursor with poor expansion and inhomogeneous composition [14]. Therefore, the formation of complex with different thermal decomposition temperatures in the solution with appropriate urea amount (i.e., U/Al) is the key to ensure the preparation of precursor with high expansion and uniform composition.

Based on the above analysis, the reaction mechanism of preparing (Al_2_O_3_ + C) precursor by the MLCS method is as follows. Firstly, the aqueous solution is heated and evaporated to form gel. Simultaneously, complexes with various amounts and decomposition temperatures are formed, and the complex formed before combustion prevents the crystallization and precipitation of aluminum nitrate, ensuring the uniform composition of Al_2_O_3_ and C mixture in the precursor. Secondly, the glucose arising from the complex, formed between glucose and aluminum nitrate, decompose, releasing a certain amount of gas and expansion of the gel along with creation of the circulation channel of gas. Thirdly, the decomposition of complexes with higher thermal stability formed among glucose, urea, and aluminum nitrate and formed between urea and aluminum nitrate, releasing a large amount of reaction gas, which triggered an exothermic reaction. Finally, a large amount of gas and heat generated expanded the gel rapidly, and triggered a self-propagating combustion, resulting in decarbonization of glucose and decomposition of aluminum nitrate, which were converted into amorphous C and Al_2_O_3_, respectively. Hence, the Al_2_O_3_ + C precursor with fluffy foam-like and high reaction activity can be obtained.

## 4. Conclusions

(1) The descending order in terms of generating various nitrogen-containing gases during combustion is N_2_, NO, N_2_O, NO_2_, N_2_O_3_, and N_2_O_4_. The type and amount of generating various nitrogen-containing gases during combustion vary with the change of U/Al.
(2) In the present experiment conditions such as ignition temperature, the decomposition reaction of aluminum nitrate is more prone to occur than the combustion reaction of urea under the concomitant condition of both.
(3) The reaction system with U/Al = 2.5 reaches the highest combustion temperature, which is consistent with the propellant chemical theory. As the value of U/Al is greater than 2.5, the combustion temperature decreases slightly due to the generated gas from the decomposition of excessive urea which carries away a part of heat.
(4) Several complexes can be formed among glucose, urea, and aluminum nitrate during heating under the combustion system. The formation of these complexes with various decomposition temperatures during combustion is the key to ensure the preparation of (Al_2_O_3_ + C) precursor with high reaction activity.

## Data Availability

The study did not report any data.

## References

[1] Kingsley J.J., Patil K.C. (1988). A novel combustion process for the synthesis of fine particle α-alumina and related materials. Mater. Lett..

[2] Wu F.L., Li X., Li Y.M., Song F.S., Wang Z.M., Shen Z.Y. (2021). Low-temperature combustion synthesis method to prepare Na+-doped ultrafine micron-sized γ-Ce_2_S_3_ bright red pigments. J. Solid State Chem..

[3] Xing S.L., Song S.L., Xiang J.K. (2020). Low temperature combustion synthesis and photoluminescence mechanism of ZnO/ZnAl_2_O_4_ composite phosphors. Optik.

[4] Qin M., Liang H.S., Zhao X.R., Wu H.J. (2020). Glycine-assisted solution combustion synthesis of NiCo_2_O_4_ electromagnetic wave absorber with wide absorption bandwidth. Ceram. Int..

[5] Zhao C.C., Yu J.C., Zhang Y.J., Gong H.Y., Xie B.Y., Lin X., Sheng M.M., Mao J.J., Jing J. (2020). Microwave-induced solution combustion synthesis and luminescent properties of nano-sized powders with different Nd concentrations. Ceram. Int..

[6] Ntola P., Friedrich H.B., Mahomed A.S., Olivier E.J., Govender A., Singh S. (2022). Exploring the role of fuel on the microstructure of VOx/MgO powders prepared using solution combustion synthesis. Mater. Chem. Phys..

[7] Patil K.C., Aruna S.T., Mimani T. (2002). Combustion synthesis: An update. Curr. Opin. Solid State Mater. Sci..

[8] Khort A., Roslyakov S., Loginov P. (2021). Solution combustion synthesis of single-phase bimetallic nanomaterials. Nanostruct.-Nanoobject..

[9] Karami M., Masoudpanah S.M., Rezaie H.R. (2021). Solution combustion synthesis of hierarchical porous LiFePO_4_ powders as cathode materials for lithium-ion batteries. Adv. Powder Technol..

[10] Chu A.M., Wang Z.Q., Rafi U.D., Dong Y.H., Guo C.G., Liu W.H., Xu H.M., Wang L. (2018). Citric acid-assisted combustion-nitridation-denitridation synthesis of well-distributed W-Cu nanocomposite powders. Int. J. Refract. Met. Hard Mater..

[11] Wang Q.Y., Wu H.Y., Qin M.L., Li Z.Y., Jia B.R., Chu A.M., Qu X.H. (2021). Study on influencing factors and mechanism of high-quality tungsten carbide nanopowders synthesized via carbothermal reduction. J. Alloys Compd..

[12] Cao Z.Q., Qin M.L., Chu A.M., Huang M., Wu H.Y., Qu X.H. (2014). Glucose-assisted combustion-nitridation synthesis of well-distributed CrN nanoparticles. Mater. Res. Bull..

[13] He Q., Qin M.L., Huang M., Chu A.M., Lu H.F., Chen P.Q., Wang H., Qu X.H. (2017). Mechanism and kinetics of combustion-carbothermal synthesis of AlN nanopowders. Ceram. Int..

[14] Chu A.M., Qin M.L., Rafi U.D., Jia B.R., Lu H.F., Qu X.H. (2012). Effect of urea on the size and morphology of AlN nanoparticles synthesized from combustion synthesis precursors. J. Alloys Compd..

[15] Chu A.M., Qin M.L., Rafi U.D., Jia B.R., Lu H.F., Qu X.H. (2012). Effect of aluminum source on the synthesis of AlN powders from combustion synthesis precursors. Mater. Res. Bull..

[16] Wu H.Y., Qin M.L., Chu A.M., Wan Q., Cao Z.Q., Liu Y., Qu X.H., Volinsky A.A. (2014). AlN powder synthesis by sodium fluoride-assisted carbothermal combustion. Ceram. Int..

[17] Frurip D.J., Syverud A.N., Chase M.W. (1985). Thermodynamic properties of diatomic gases at high temperatures: An improved calculational approach for the JANAF thermochemical tables. J. Nucl. Mater..

[18] Pacewska B., Keshr M. (2002). Thermal transformations of aluminium nitrate hydrate. Thermochim. Acta.

[19] Biamino S., Badini C. (2004). Combustion synthesis of lanthanum chromite starting from water solutions: Investigation of process mechanism by DTA-TGA-MS. J. Eur. Ceram. Soc..

[20] Fumo D.A., More M.R., Segadaes A.M. (1996). Combustion synthesis of calcium aluminates. Mater. Res. Bull..

[21] Zhang Y., Stangle G.C. (1994). Preparation of fine multicomponent oxide ceramic powder by a combustion synthesis process. J. Mater. Res..

[22] Chandramouli V., Anthonysamy S., Rao P.R.V. (1999). Combustion synthesis of thoria-a feasibility study. J. Nucl. Mater..

[23] Kim E.S., Yaylayan V. (2021). Identification of the Maillard reaction intermediates as divalent iron complexes in alanine/glucose/FeCl_2_ model system using ESI/TOF/MS/MS and isotope labelling technique. Curr. Res. Food. Sci..

[24] Korolevich M.V., Zhbankov R.G., Sivchik V.V. (1990). Calculation of absorption band frequencies and intensities in the IR spectrum of α-d-glucose in a cluster. J. Mol. Struct..

